# A New Method for* In Vivo* Analysis of the Performances of a Heat and Moisture Exchanger (HME) in Mechanically Ventilated Patients

**DOI:** 10.1155/2019/9270615

**Published:** 2019-02-26

**Authors:** Matteo Filippini, Mauro Serpelloni, Valeria Quaranta, Paolo Bellitti, Emilio Sardini, Nicola Latronico

**Affiliations:** ^1^Department of Anaesthesia and Critical Care Medicine, University of Brescia at Spedali Civili di Brescia, 25100 Brescia, Italy; ^2^Department of Information Engineering, University of Brescia, 25123 Brescia, Italy; ^3^Department of Medical and Surgical Specialties, Radiological Sciences and Public Health, Italy

## Abstract

**Aim:**

To evaluate the conditioning capabilities of the DAR™ Hygrobac™ S, a Heat and Moisture Exchanger (HME), using a new device to measure the temperature (T) and the absolute humidity (AH) of the ventilated gases* in vivo* during mechanical ventilation in Intensive Care Unit (ICU) patients.

**Materials and Methods:**

In 49 mechanically ventilated ICU patients, we evaluated T and AH, indicating the HME efficacy, during the inspiratory phase upstream and downstream the HME and the ratio of inspired AH to expired AH and the difference between expired T and inspired T indicated the HME efficiency. Efficacy and efficiency were assessed at three time points: at baseline (t_0_, HME positioning time), at 12 hours (t_1_), and at 24 hours (t_2_) using a dedicated,* ad hoc* built wireless device. Differences over time were evaluated using one-way ANOVA for repeated measures, whereas differences between* in vivo* and laboratory values (declared by the manufacturer according to UNI® EN ISO 9360 international standard) were evaluated using one-sample Student t-test.

**Results:**

49 HMEs were analysed* in vivo* during mechanical ventilation. T and AH means (SD) of the inspired gas (the efficacy) were 31.5°C (1.54) and 32.3 mg/l (2.60) at t_0_, 31.1°C (1.34) and 31.7 mg/l (2.26) at t_1_, and 31°C (1.29) and 31.4 mg/l (2.27) at t_2_. Both efficiency parameters were constant over time (inspired AH/expired AH=89%, p=0.24; and expired T–inspired T = 2.2°C, p=0.81). Compared with laboratory values,* in vivo* T and AH indicating efficacy were significantly lower (p<0.01), whereas the efficiency was significantly higher (p<0.01).

**Conclusions:**

HME performances can be accurately assessed for prolonged periods* in vivo* during routine mechanical ventilation in ICU patients. Temperature and absolute humidity of ventilated gases* in vivo* were maintained within the expected range and remained stable over time. HME efficacy and efficiency* in vivo* significantly differed from laboratory values.

## 1. Introduction

Conditioning (heating and humidifying) of inspired gases is one of the functions of the upper airways. In critically ill patients, tracheal intubation or tracheostomy limits the efficacy of this process; therefore, when gases reach the lower airways, they are inadequately humidified and heated. This inadequate humidification may lead to respiratory heat loss, airway obstruction due to thick secretions, and impairment of the mucociliary function. Heat and Moisture Exchangers (HMEs) combined with microbiological filters are currently recommended to reduce the risk of respiratory complications caused by inadequate warming and humidification of the gases delivered to mechanically ventilated patients [1–9].

The performance of HMEs is dependent upon the type of device, the ventilator setting (i.e., minute ventilation, and tidal volume), and room and patient temperatures. Based on normal physiology, HMEs should be able to provide 30 to 33 mg/l of water to the airways at 30-36°C for optimal use in the Intensive Care Unit (ICU) [10, 11]. The commercially available devices are tested in the laboratory according to UNI® EN ISO 9360 international standard because the* in vivo* measurements (during mechanical ventilation) of the humidity and temperature are technically demanding [12–16]. Recently, some* in vivo* methods have been developed; such methods enable the measurement of water exchange performance of a variety of HMEs within a short timeframe [17, 18].

In this study, we applied a new method for a rapid and noninvasive assessment of the HME performance during mechanical ventilation. The main characteristic of this method was the use of a new device which can also be applied for long-term assessment of the HME performance. We measured the conditioning capabilities of a commercially available HME and its performance variations over time during a 24-hour period of mechanical ventilation with continuous HME use in a widely heterogeneous population of artificially ventilated ICU patients.

## 2. Materials and Methods

This was a retrospective analysis of prospectively collected data from November 2016 to August 2017 at the general ICU of the Spedali Civili, a regional hospital, affiliated with the University of Brescia, Italy. We enrolled 49 critically ill patients who had been mechanically ventilated for at least 24 hours with use of a HME to provide adequate passive gas conditioning. These patients were studied with a new device, built by researchers of the Department of Information Engineering of the University of Brescia (MS, PB, and ES) to measure the performances of the DAR™ Hygrobac™ S, a HME of standard clinical use at the time of the study.

The study was approved by the Ethics Committee of the University of Brescia at Spedali Civili (protocol number 2440, June 20th, 2016) that waived the requirement for consent because the study only involved recording data from the medical device with complete patients anonymisation (i.e., the data subjects were not identifiable).

The HMEs were changed routinely every 24 hours as per manufacturer's recommendations or earlier in case of copious secretions or when increases in airway pressure were thought to be due to increased HME resistance. The ventilator circuit remained intact for the duration of the period of ventilation. The ventilatory settings were not modified during the 24-hour study period (unless required by clinical conditions, but never significantly). The incharge intensivist was not directly involved in the study and took full responsibility for clinical and ventilatory management.

### 2.1. Description of the Experiment and Definitions

In this study, we defined the following HME performance indicators:

- **Input**: as the temperature (T) and absolute humidity (AH) of fresh gases flowing from the ventilator to the HME during inspiration;
- **Load**: as the T and AH of the gases flowing from the patient to the HME during expiration;
- **Return**: as the T and AH of gases flowing from the HME to the patient during inspiration (it describes the** “efficacy”**);
- **Loss**: as the T and AH of gases leaving the HME for the ventilator during expiration;
- **Yield**: as the* AH return *to* AH load* ratio (it describes the** “efficiency”**);
- **Thermal differential**: as the difference between T* load* and T* return *(it describes the** “efficiency”**);

T and AH values were measured by means of the sensors described below.

Data were recorded in three different moments:

- at HME positioning time** [t**_**0**_**]**
- after 12 hours of use** [t**_**1**_**]**
- at the end of 24 hours of use** [t**_**2**_**]**

Tidal volumes, respiratory rates, minute ventilation, inspiration:expiration time (I:E) ratios, and room and patient temperatures were also recorded in those moments.

We assessed the following: (a) the absolute T and AH values of input, load, return, loss, yield, and thermal differential; (b) their variation over time (24 hours); and (c) the differences between* in vivo* obtained values of HME performance indicators and their laboratory values declared by the manufacturer in the technical sheet and/or prescribed by the UNI® EN ISO 9360 international standard. In particular, T and AH declared laboratory values (efficacy) were 32.3°C and 33.6 mg/l, respectively, whereas efficiency laboratory values (inspired AH/expired AH and expired–inspired T) were 77% and 4.7°C, respectively.

### 2.2. Description of the Instrument

The measurement system was composed of two main sections, called* Measuring Section* and* Reading Section*. The first was the core of the project and it executed the main operations: reading the sensors, building the data block, and managing the Bluetooth® Low Energy (BLE) connection; this was also subdivided into two parts: machine side and patient side.

**Figure 1:**
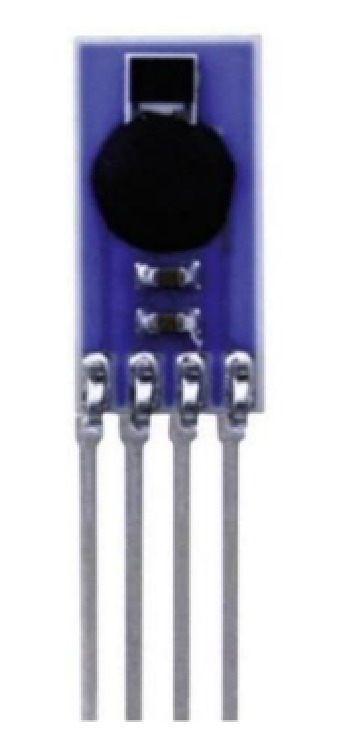
T and AH sensor: IST® HYT 271 (machine side).

**Figure 2:**
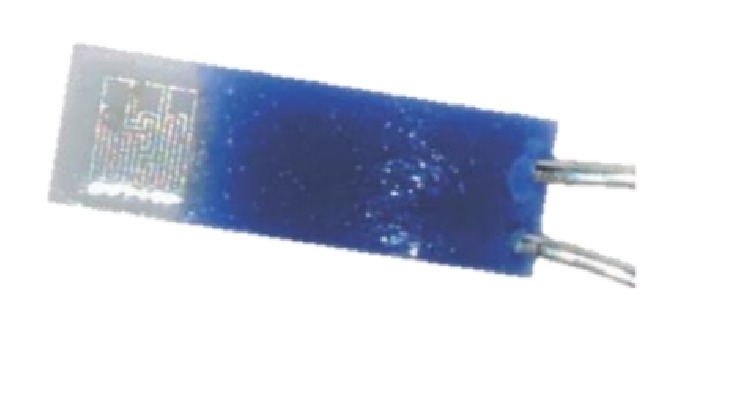
Tsensor: RTD HERAEUS® M222 Pt1000 (patient side).

**Figure 3:**
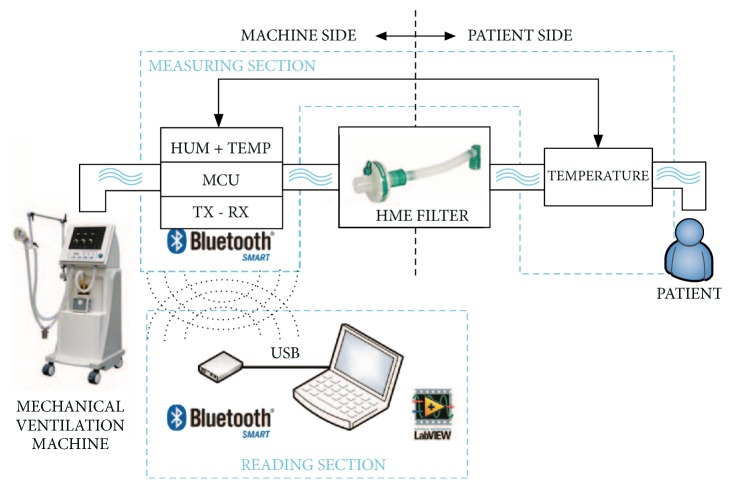
Data acquisition system.

Project specifications required two different measurement points, upstream and downstream the HME filter. Upstream the HME (machine side), T and relative humidity (RH) were measured using the IST® HYT 271 sensor (Figure 1) and downstream that (patient side) only T using the RTD HERAEUS® M222 Pt1000 sensor (Figure 2), considering that on the patient's side the air is always almost saturated in water content (RH 100%) during expiration and it is about RH 98% during inspiration [21, 22]. The* Reading Section* read the data sent by Measuring Section and converted the raw data to T and AH values by adopting the formulas reported elsewhere [19] (Figure 3).

The complete system, measuring module (sensors and electronics) and display module, was validated in the laboratory before clinical trials. After an evaluation in the climatic chamber, a specific analysis of the dynamic behaviour of the humidity and temperature sensors has been performed, including a comparison of the measured data to typical clinical T and AH variation to assess error in the evaluation of T and AH values. The results showed that the percentage error of the measured values compared to the real signals was from 5% to 14% depending on respiratory frequency rate and I:E ratio, so the error trend was linear with the increase of the respiratory frequency rate and was inversely proportional to the I:E ratio [23–30].

### 2.3. Data Presentation and Statistical Analysis

All variables were expressed as means and standard deviations (SD).

We estimated that a sample of 36 HMEs would provide 80% power to detect a difference of 2°C in T, 2 mg/l of AH, and a 5% variation in the yield, at a two-sided alpha level of 0.05.

Variations over time of the HME performance indicators (input, load, return, loss, yield, and thermal differential) were analysed using ANOVA for repeated measures. The differences between* in vivo* observed and manufacturer-declared laboratory values were analysed using one-sample Student t-test. A p < 0.05 was considered as statistically significant.

## 3. Results

We evaluated 49 HMEs* in vivo* in 49 mechanically ventilated ICU patients. For 36 HMEs, the performances were analysed for 24 hours according to the study protocol, while for 13 HMEs the assessment was interrupted after 12 hours because of device replacement for clinical needs.

Table 1 describes the ventilatory variables, as well as room and patient temperatures at t_0_, t_1_, and t_2_. The minute ventilation varied between 7.42 and 7.52 l/min, room temperature between 23 to 24°C, and patient temperature between 36.7 and 36.9°C. There were no statistically significant changes in these parameters over time.

Concerning the main aims of the study, (a) T and AH of the inspired gases, describing the efficacy, are shown in Table 2, (b) none of the measured parameters significantly varied over time (Table 2, last column), and (c) differences between* in vivo* observed and laboratory values are shown in Table 3.

*In vivo* measured efficacy was lower than the laboratory values for both T and AH, whereas the efficiency was higher* in vivo* than in laboratory conditions (p < 0.01). Moreover, our clinical setting was significantly different in terms of tidal volumes, respiratory rates, minute ventilations, I:E ratios, room temperatures, patient temperatures, and load T and AH, compared to the laboratory setting (Table 3).

## 4. Discussion

In this retrospective study, we analysed the DAR™ Hygrobac™ S HME performances* in vivo* during routine mechanical ventilation in critically ill patients by applying a newly devised wireless, portable device of reduced weight, with a user-friendly interface and long battery life, measuring the gas temperature and absolute humidity with low invasiveness for the patients and complete integration with respiratory circuit. By using a complete list of HME performance indicators, we found that the* in vivo* efficacy of the HME was within the required range for optimal gas conditioning which should be maintained between 30 and 36°C of T and 30 and 33 mg/l of AH and did not significantly change over time. The HME* in vivo* efficacy was lower than expected based on laboratory values; i.e., the HME provided less heat and humidity than anticipated; on the contrary, the efficiency was higher; i.e., the ratio of AH return to load was 89%, which was significantly higher than the 77% expected value based on laboratory values. The reasons for differences in efficacy and efficiency between* in vivo* and laboratory values are speculative. Other factors, in addition to the HME itself, may have influenced these parameters. The patient's and room temperature, the type and the length of the tracheal tube, and the ventilatory setting might have played a role; however, the evidence supporting this hypothesis has not been evaluated in other studies. Importantly, these ambient factors are an integral part of* in vivo* functioning of the HME and should be assessed in future studies aiming at quantifying their impact on HME performances.

## 5. Conclusions

In conclusion, we demonstrated that HME performances can be accurately assessed for prolonged periods* in vivo* during routine mechanical ventilation in ICU patients with the use of a newly introduced device. Temperature and absolute humidity of ventilated gases* in vivo* were maintained within the expected range and remained stable over the entire observation period. However, HME efficacy and efficiency* in vivo* differed significantly from laboratory values.

## Figures and Tables

**Table 1:** Ventilatory variables at t_0_, t_1_, and t_2_ assessment time; SD: standard deviation.

	t_0_: mean	t_1_: mean	t_2_: mean	Differences over time (p)
	(SD)	(SD)	(SD)	
Tidal volume (ml)	443	431	425	0.70
	(136)	(109)	(93)	

Respiratory rate (min^−1^)	18	18	18	0.82
	(6.55)	(6.91)	(6.91)	

Minute ventilation (l/min)	7.46	7.52	7.42	0.92
	(2.49)	(2.13)	(2.41)	

I:E ratio	0.53	0.59	0.58	0.70
	(0.26)	(0.27)	(0.28)	

Room temperature (°C)	24.0	23.3	23.7	0.09
	(1.75)	(1.14)	(1.16)	

Patient temperature (°C)	36.7	36.9	36.7	0.76
	(0.70)	(0.75)	(0.59)	

**Table 2:** Temperature (T) and absolute humidity (AH) values of Heat and Moisture Exchanger (HME) performance indicators; SD: standard deviation.

HME performance indicators		t_0_: mean	t_1_: mean	t_2_: mean	Differences over time (p)
		(SD)	(SD)	(SD)	
LOAD	T (°C)	33.6	33.4	33.3	0.51
		(1.38)	(1.49)	(1.18)	
	AH (mg/l)	36.9	36.4	36.2	0.18
		(2.66)	(2.83)	(2.26)	

RETURN (efficacy)	T (°C)	31.5	31.1	31.0	0.67
		(1.54)	(1.34)	(1.29)	
	AH (mg/l)	32.3	31.7	31.4	0.11
		(2.60)	(2.26)	(2.27)	

YIELD (efficiency)	Thermal differential (°C)	2.2	2.2	2.3	0.81
		(0.55)	(0.54)	(0.76)	
	AH yield	0.89	0.89	0.89	0.24
		(0.03)	(0.03)	(0.04)	

INPUT	T (°C)	25.9	25.2	25.5	0.80
		(0.76)	(0.71)	(0.88)	
	AH (mg/l)	1.2	1.3	1.2	0.11
		(0.70)	(0.52)	(0.56)	

LOSS	T (°C)	26.0	25.3	25.5	0.59
		(0.76)	(0.72)	(0.89)	
	AH (mg/l)	6.3	6.9	6.6	0.62
		(1.36)	(1.94)	(2.57)	

**Table 3:** Comparison of temperature (T) and absolute humidity (AH) of Heat and Moisture Exchanger (HME) performance indicators between laboratory values as declared by the manufacturer and *in vivo* measured values. All values are expressed as laboratory values minus *in vivo* values and were measured at a tidal volume of 500 ml.

HME performance indicators and ventilatory parameters	*Laboratory* – *in vivo* t_0_	*Laboratory* – *in vivo* t_1_	*Laboratory* – *in vivo* t_2_	*Laboratory* values [15, 16, 19, 20]
Load T (°C)	+ 3.4	+ 3.7	+ 3.7	37.0
	(p < 0.01)	(p < 0.01)	(p < 0.01)	

Load AH (mg/l)	+ 6.9	+ 7.4	+ 7.6	43.8
	(p < 0.01)	(p < 0.01)	(p < 0.01)	

Return T (efficacy) (°C)	+ 0.8	+ 1.2	+ 1.3	32.3
	(p < 0.01)	(p < 0.01)	(p < 0.01)	

Return AH (efficacy) (mg/l)	+ 1.3	+ 2.0	+ 2.2	33.6
	(p < 0.01)	(p < 0.01)	(p < 0.01)	

Thermal differential (efficiency) (°C)	+ 2.5	+ 2.5	+ 2.4	4.7
	(p < 0.01)	(p < 0.01)	(p < 0.01)	

AH yield (efficiency)	- 0.12	- 0.12	- 0.12	0.77
	(p < 0.01)	(p < 0.01)	(p < 0.01)	

Input T (°C)	- 2.9	- 2.2	- 2.5	23.0
	(p < 0.01)	(p < 0.01)	(p < 0.01)	

Input AH (mg/l)	- 0.2	- 0.3	- 0.2	1.0
	(p = 0.03)	(p < 0.01)	(p = 0.049)	

Loss T (°C)	- 3.0	- 2.3	- 2.5	23.0
	(p < 0.01)	(p < 0.01)	(p < 0.01)	

Loss AH (mg/l)	- 0.3	- 0.9	- 0.6	6.0
	(p = 0.10)	(p <0.01)	(p =0.16)	

Tidal volume (ml)	+ 57	+ 69	+ 75	500
	(p < 0.01)	(p < 0.01)	(p < 0.01)	

Respiratory rate (min^−1^)	- 3	- 3	- 3	15
	(p < 0.01)	(p < 0.01)	(p < 0.01)	

Minute ventilation (l/min)	+ 0.04	- 0.02	+ 0.08	7.5
	(p = 0.91)	(p = 0.96)	(p = 0.84)	

I:E ratio	+ 0.47	+ 0.41	+ 0.42	1
	(p < 0.01)	(p < 0.01)	(p < 0.01)	

Room temperature (°C)	- 1.0	- 0.3	- 0.7	23.0
	(p < 0.01)	(p = 0.63)	(p < 0.01)	

Patient temperature (°C)	+ 0.4	+ 0.1	+ 0.3	37.0
	(p < 0.01)	(p = 0.12)	(p < 0.01)	

## Data Availability

All the data used to support the findings of this study are available from the corresponding author upon request.
